# Diabetes in Pregnant Romanian Patients—Epidemiology and Prevention Strategies Proposal

**DOI:** 10.3390/jcm14228135

**Published:** 2025-11-17

**Authors:** Bianca-Margareta Salmen, Teodor Salmen, Delia Reurean-Pintilei, Cristina Vaida, Roxana-Elena Bohiltea

**Affiliations:** 1 Doctoral School, ‘Carol Davila’ University of Medicine and Pharmacy, 050474 Bucharest, Romania; bianca-margareta.mihai@drd.umfcd.ro; 2 Pitesti Emergency County Hospital, 110283 Pitesti, Romania; 3 Department of Medical-Surgical and Complementary Sciences, Faculty of Medicine and Biological Sciences, “Ștefan cel Mare” University, 720229 Suceava, Romania; delia.pintilei@usm.ro; 4 Department of Diabetes, Nutrition and Metabolic Diseases, Consultmed Medical Centre, 700544 Iasi, Romania; 5 Department of Physical Medicine and Rehabilitation, Altstadtpraxis Aarau GmbH, 5000 Aarau, Switzerland; cristina.vaida@bluewin.ch; 6 Department of Obstetrics and Gynaecology, ‘Carol Davila’ University of Medicine and Pharmacy, 050474 Bucharest, Romania; roxana.bohiltea@umfcd.ro

**Keywords:** diabetes mellitus in pregnancy, Romania, gestational diabetes, epidemiology, vulnerable pregnancies, social issues

## Abstract

**Background/Objectives**: Diabetes mellitus (DM) in pregnancy, including type 1 (T1DM), type 2 (T2DM), and gestational DM (GDM), represents an increasing health burden due to its maternal and fetal complications. Despite the increment in the global prevalence estimates of DM in pregnancy, in Romania, it has not been comprehensively described. This study aimed to analyze the prevalence and dynamics of DM in pregnancy in Romania between 2014 and 2024, using national databases, and to identify prevention strategies for reducing maternal and fetal complications. **Methods**: Data were obtained from the Romanian National Public Health Institute through two distinct sources: Database 1, consisting of reports from public and medical units associated with the National Health Insurance House and Database 2, based on the reports from general practitioners. Pregnancies complicated by DM were assessed by type, age group, and environmental settlement. Additional data were extracted on pregnancies with insufficient prenatal care and those of socially vulnerable individuals. **Results**: From 2014 to 2024, the prevalence of DM in pregnancy in Romania was consistently lower than European and global estimates, ranging from 1.01‰ to 3.08‰ in Database 1 and from 0.84‰ to 5.88‰ in Database 2, respectively. GDM accounted for the majority of cases, accounting for 65–88% of reported DM in pregnancy. The highest incidence was observed in the 20–39 years age group, with a growing proportion in women aged ≥40 years. Urban-rural disparities decreased over the decade, with rural cases reaching parity by 2024. Vulnerable populations included adolescents, women with insufficient prenatal care, and those with social risk factors, predominantly from rural areas. **Conclusions**: Although the reported prevalence of DM in pregnancy in Romania is lower than international figures, the true burden is likely underestimated. GDM remains the leading type of DM in pregnancy, mirroring global trends. Strengthening the reporting system, standardizing diagnostic criteria, and targeting high-risk groups through preconceptional counselling, lifestyle interventions, advanced monitoring technologies, and improving social support through the involved authorities are crucial steps to reduce maternal and fetal morbidity.

## 1. Introduction

Diabetes mellitus (DM) is the term used for a metabolic disorder group in which carbohydrate metabolism is altered, resulting in a chronic hyperglycemic status [1]. This group includes type 1 DM (T1DM), type 2 DM (T2DM), specific forms of DM due to specific causes (i.e., exocrine pancreas diseases, monogenic syndromes, chemical or drug-induced DM) and gestational DM (GDM) [1,2]. DM represents an important health issue, as its incidence has an increasing trend worldwide due to the obesity pandemic. It affects more and more young adults, including females of childbearing age [3].

In conditions of poor glycemic control, pregestational DM and GDM lead to well-known vascular complications, the most frequent being cardiovascular (CV) disease, ocular, vascular, renal, and neurologic pathologies. Also, they are associated with short-term and long-term morbidities that affect both the mother and the fetus. Among short-term morbidities, we encounter preterm birth, gestational hypertension, preeclampsia [4], large for gestational age fetuses and macrosomia [5], polyhydramnios [6], intrauterine growth restriction [7], neonatal complications (respiratory distress syndrome, cardiomyopathy, hypoglycemia, hypomagnesemia, hypocalcemia or hyperbilirubinemia) [8] or even stillbirth [9]. Furthermore, due to hyperglycemia in the first trimester and periconceptional period, pregestational DM is associated with a two-to-four-fold higher risk of congenital malformations: congenital heart disease (transposition of the great arteries, tetralogy of Fallot, anomalous venous return or septal defects) [10], central nervous malformations (spina bifida, anencephaly, hydrocephaly, microtia or anotia, encephalocel) [11] and sacral or caudal dysplasia [12].

During the peripartum period, newborns of diabetic mothers are at a higher risk of shoulder dystocia, brachial plexus palsy or clavicular fracture, independently of the birth weight [13]. After childbirth, mothers with GDM present a higher risk of developing metabolic syndrome in the first three months [14], as well as a nearly tenfold higher risk of developing T2DM in the course of time [15]. Additionally, they have a twofold increased risk of developing CV disease compared to healthy females, independently of the presence of T2DM [16]. Long-term complications for the offspring include a future risk for developing obesity [17], T1DM or T2DM [18]. Regarding long-term complications, infants from diabetic mothers are at high risk of becoming obese [19], as well as developing metabolic syndrome, T1DM or T2DM in the future [20].

Due to the constantly expanding incidence of DM, it is difficult to quantify the exact number of cases. A study published by Jovanovič et al. [21] in 2015 concluded that the incidence of DM in pregnancy in the United States was 7.9% in their study period (2005–2011). Of those patients, 0.1% had T1DM, 1.2% had T2DM, 6.3% had GDM, and 0.2% had progressing GDM. Another study reported that, in 2020, the prevalence of GDM varied from 2% to 38% in other countries [22], while in the United States, it was 7.8% [23].

Considering the alarmingly rising number of DM cases during pregnancy as well as the burden that DM during pregnancy places on the healthcare system, mothers and infants, this article aims to report DM in pregnancy incidence numbers and analyze the dynamics in Romania, as well as propose prevention strategies for pregnancies complicated by GDM or T2DM.

## 2. Materials and Methods

### 2.1. Study Design

We performed a retrospective analysis using reports from the Romanian National Public Health Institute. These reports include the total number of pregnancies, the total number of pregnancies involving patients with diabetes mellitus (DM), and the distribution of these pregnancies by DM type and environmental settlement. The reports also include the number of cases involving pregnant patients who received insufficient prenatal care, as well as the number of socially vulnerable patients reported in Romania between 2014 and 2024 [24].

### 2.2. Study Population

Our study includes reports on Romanian DM complicated pregnancies between 2014 and 2024. DM type diagnosis was performed according to the national medical practice [25]. GDM diagnosis was realized after performing the 75 g oral glucose tolerance test at 24–28 gestational weeks using venous blood samples; the diagnosis was made when any of the following plasma glucose values were achieved or exceeded: a 92 mg/dL value of the fasting plasma glucose, a 180 mg/dL value of plasma glucose at one hour after the ingestion of 75 g glucose, or a value of at least 153 mg/dL at two hours after the glucose ingestion. Pregestational diabetes was diagnosed based on the American Diabetes Association (ADA) criteria [2]: a fasting plasma glucose value that meets or exceeds 126 mg/dL or a two-hour plasma glucose value of at least 200 mg/dL during the oral glucose tolerance test or a glycated hemoglobin (HbA1c) value of at least 6.5%.

### 2.3. Data Collection

We obtained two databases from two distinct sources: Database 1 consists of data reported by public and private medical units associated with the National Health Insurance House (according to the Health Minister Order no 1782/2006), and Database 2 consists of data collected by the National Public Health Department from the general practitioners’ offices. The data was reported to the National Public Health Department using the International Classification of Diseases (ICD) codification. The two databases cannot be merged because the same patient can be reported in both databases; therefore, we will analyze the two sources separately. It is important to emphasize that all pregnant women are requested to schedule regular appointments with their general practitioner for pregnancy follow-up, so their reports are more likely to include almost all pregnant women.

### 2.4. Statistical Analysis

The databases are reported as Microsoft Excel databases and include reports in the form of counts and percentages. We performed statistical analyses using the Microsoft Excel Version 2021, and Statistical Package for Social Sciences (SPSS), Version 20. We calculated prevalences with their confidence intervals (CIs), Pearson’s chi-square, Spearman’s test for correlations, and Cronbach’s alpha for reliability analysis. A p-value lower than 0.05 was considered statistically significant for differences between the variables from the Gaussian distribution.

## 3. Results

### Epidemiology of Pregnancies Complicated with DM in Romania

Table 1 shows the total number of pregnancies from Romania between 2014 and 2024, as well as the number of pregnancies from Romania complicated by DM and how patients are distributed by the type of DM.

When analyzing the prevalence of DM in pregnancy in this eleven-year period, we obtained a statistically significant correlation (Spearman’s correlation *r* = 0.663, *p* for trend 0.026).

**Figure 1:**
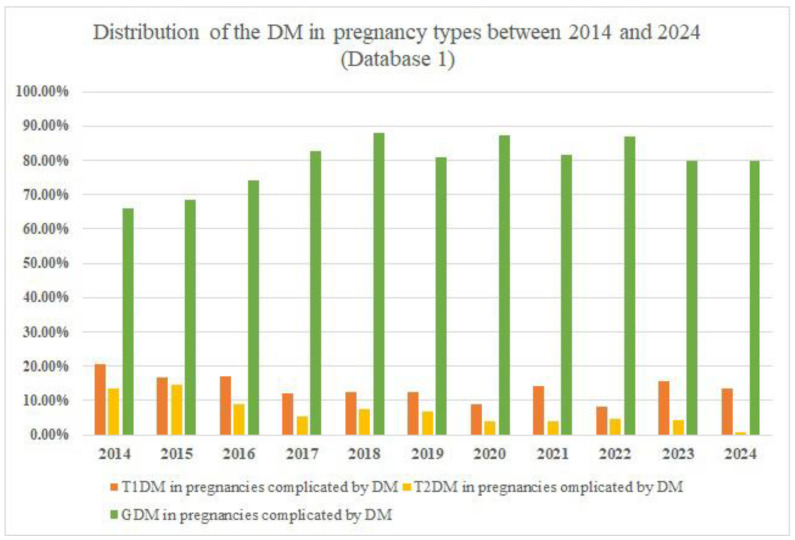
DM in pregnancy types distribution between 2014 and 2024, as reported by Database 1.

Figure 1 shows the proportions of the DM in pregnancy types, as reported by Database 1.

Table 2 reports the distribution of DM in pregnant women in Romania between 2014 and 2024, classified by age group (10–14 years, 15–19 years, 20–39 years and 40–54 years) as reported by Database 2.

The data in Table 3 shows the distribution of DM cases in pregnancies in Romania between 2014 and 2024, categorized by environmental settlement, as reported by Database 1.

**Figure 2:**
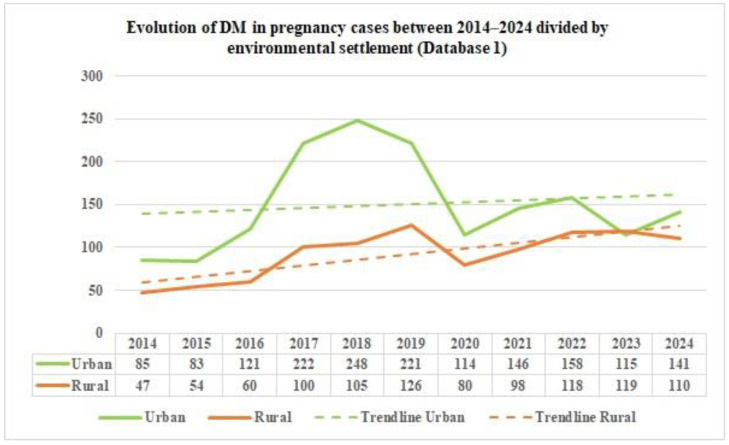
The evolution and trend of the DM in pregnancy cases between 2014 and 2024, divided by environmental settlement, as reported by Database 1.

As shown in Figure 2, there is an upward trend, with a steeper rise in the DM cases during pregnancy in the urban areas compared to the rural areas during the period of interest, as reported by Database 1. However, although there is a slight increase in the cases of DM in urban areas, there are unexpected temporal modifications (especially prevalence peaks) that could be explained by social, economic or demographic factors.

Table 4 represents the distribution of cases of DM in pregnancies from Romania between 2014 and 2024 after the environmental settlement as reported by the general practitioners in Database 2.

**Figure 3:**
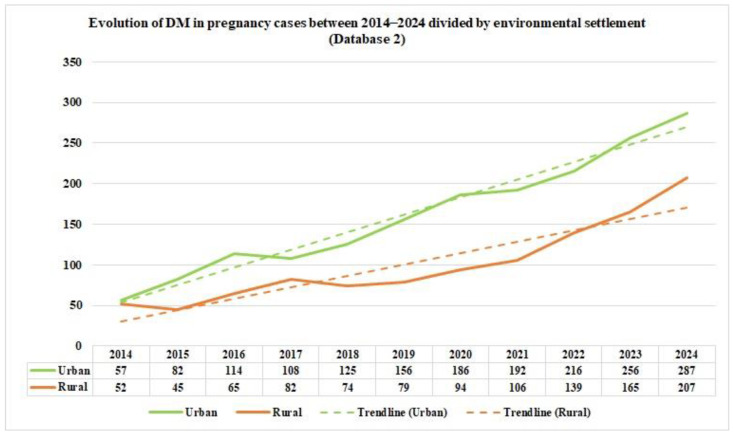
The evolution and trend of the DM in pregnancy cases between 2014 and 2024, divided by environmental settlement, as reported by Database 2.

Following the data reported by Database 2, DM in pregnancy shows an upward trend, with slight temporal modifications in the prevalence percentages. This upward trend is more obvious in the urban areas (Figure 3).

Following the Cronbach’s alpha reliability analysis between the two databases, regarding the reported number of DM in pregnancy, we obtained an α value of 0.371, a value that can be influenced by the small number of analyzed scales.

Table 5 illustrates the DM in pregnancy cases in Romania between 2014 and 2024, categorized by age group (10–19, 20–39 and 40–49 years), as documented in Database 1.

Table 6 represents the distribution of cases of pregnancies from Romania between 2014 and 2024 that received insufficient prenatal care and were socially vulnerable patients, as reported in Database 1.

Table 7 represents the distribution of cases of pregnancies from Romania between 2014 and 2024 that received insufficient prenatal care and socially vulnerable patients by environmental settlement, as reported in Database 1.

We analyzed the association between risk factors such as environmental settlement, socially vulnerable patients, patients who received insufficient prenatal care, the total number of pregnancies, the total number of DM in pregnancy, the number of cases of T1DM in pregnancy, T2DM in pregnancy, and GDM, and performed Spearman’s correlations. As shown in Table 8, we found statistically significant correlations (*p* < 0.05) between urban areas and the total number of DM and T1DM cases in pregnancy and GDM. Significant correlations were also found between rural areas and the total number of pregnancies and the total number of DM in pregnancy.

## 4. Discussion

This study aimed to analyze the data reported by the Romanian National Public Health Institute in order to provide a comprehensive overview of the incidence of DM in Romanian pregnancies between 2014 and 2024. This analysis was undertaken due to the alarming rise in GDM incidence, which has placed a significant strain on the healthcare system. Two databases were obtained for this analysis. Database 1 was reported by medical units from the public and private sectors associated with the National Health Insurance House (according to the Health Minister Order no 1782/2006). Database 2, reported by the general practitioner to the Public Health Department. However, these databases cannot be merged due to the risk of overreporting. Combining these databases provides a clear view of the situation, enabling analysis of its dynamics and the development of prevention strategies for pregnancies complicated by GDM, T1DM, or T2DM. This is especially important because the data includes information on patients’ place of residence and risk category. It is important to note that, in light of national regulatory frameworks, Database 2 is the most likely to report more accurate incidence rates.

An initial observation of the data reported in Database 1, which relates to the annual incidence of reported pregnancies in Romania, reveals a worrying downward trend in the number of pregnant patients over the period in question. This phenomenon aligns with the findings of the 2021 Global Burden of Disease Study [26], which provides an interpretation of the data. The study indicates a global decline in fertility characterized by varying degrees of steepness in the downward trend of livebirths commencing in 2000. The study’s global forecast is an enigmatic one, encompassing persistently declining fertility rates. Additionally, an analysis of the distribution of DM in pregnancy shows that reported rates were lower from 2014 to 2024 than those published in the literature.

Data from Database 1 reveal that the prevalence of DM in pregnancy ranged from approximately 1.01 to 3.08 per 1000 (95% CI spanning roughly from 1.00 to 3.20) between 2014 and 2024. Meanwhile, data from Database 2 reveal a prevalence of DM in pregnancy ranging from 0.84‰ to 5.88‰ (95% CI spanning from 0.82‰ to 5.95‰) over the same period, indicating a consistently increasing trend. The prevalence of pregestational DM in Romania between 2014 and 2024 is approximately 0.45‰ (95% CI 0.44‰–0.46‰). A systematic review and meta-analysis of the existing literature [27] reports an overall prevalence of pregestational DM between 2011 and 2020 of 1.0% (95% CI 0.6–1.5), while the pooled prevalence in Europe was 0.5% (95% CI 0.4–0.7, *p* < 0.01). A systematic review and meta-analysis conducted by Paulo et al. [28] concerning the prevalence of GDM in Europe between 2014 and 2019, concluded that the mean prevalence of GDM in Europe is approximately 11%, reaching a higher prevalence of around 31.5% in Eastern European countries. The overall prevalence of pregnancies complicated by GDM in Romania from 2014 to 2024 is approximately 1.86‰ (95% CI 1.84–1.89), with the highest prevalence being reported in 2019 at 2.56‰ (95% CI 2.53–2.58). This difference can be explained by a deficient reporting system from the medical units based on the incomplete codification of the diagnosis.

The rate of pregnancies complicated by DM peaked in 2018 with 353 reported cases. Another observation is represented by the fact that GDM accounts for at least two-thirds of all cases of DM in pregnancy. A study published in 2022 [29] also reported a division between pregestational DM and GDM, with GDM accounting for the majority of cases. The study examined the population of Belgrade, Serbia, between 2010 and 2020 and found that the prevalence of DM in pregnancy was 3.4%, with GDM accounting for 2.7% of cases and pregestational DM accounting for 0.7%. Additionally, a study conducted on the German population between 2013 and 2019 and published in 2023 [30] reported a GDM prevalence of 5.7%, with a much lower pregestational DM prevalence of 0.93%. Another important fact is that many cases could be misdiagnosed as GDM instead of pregestational DM due to the lack of medical history, preconceptional screening for DM, or the homogeneity of the GDM diagnosis method. The correct diagnosis becomes clear after birth. This issue is persistent worldwide, as there is no consensus on the GDM diagnosis method. For example, ADA presents two methods of GDM diagnosis: the one-step and two-step methods [2]. In contrast, the Australasian Diabetes in Pregnancy Society recommends using only the 75 g oral glucose tolerance test [31].

From the data on the distribution of DM in pregnancy cases in Romania by age group, as reported by Database 2, in the study period, we can notice that the majority of DM in pregnancy cases occurred in the 20–39 age group during the study period. Furthermore, based on the data from the two distinct databases, we can emphasize the fact that there is a significant difference in the reported data starting in 2017. This is because the number of cases in the Database has almost doubled; this can be explained by the exclusion of general practitioners from pregnancy monitoring. From 2020 onwards, the number of cases in Database 1 is lower, suggesting either fewer hospitalizations of pregnant patients with DM or deficient reporting by medical units. Regarding environmental settlement, analyzing the number of cases of DM in pregnancy from Romania between 2014 and 2024, as reported in Database 1, reveals a higher prevalence in the urban areas in 2014, at around two-thirds, suggesting urban–rural disparities. After eleven years, the rates in the rural and urban areas equalized.

This homogenization between areas could be due to many factors, such as better accessibility to health services and diagnosis in the rural areas, decreased fear or increased awareness of the need for medical appointments and pregnancy monitoring, or, in the worst-case scenario, a decrease in food quality intake and an increase in unhealthy diets and lifestyles in the rural areas. In contrast, the data concerning the environmental settlement of the DM in pregnancy cases from Romania between 2014 and 2024, as reported in Database 2, shows an almost equalization of the cases of DM in pregnancy in rural and urban areas. From 2017 to 2024, there was a higher prevalence of approximately two-thirds in the urban areas. A study conducted on Moroccan pregnant patients with GDM, conducted between October 2018 and February 2019 [32], found no statistically significant difference in environmental settlement (*p* = 0.171) and reported 52.9% cases from the rural areas and 47.1% cases belonging to the urban areas. In contrast, a study published in 2023 [33], conducted between 2011 and 2019 in the United States, analyzing 12,401,888 singleton live births from nulliparous females, concluded that patients living in the rural areas present an increased overall risk of developing pregestational DM, with an age-adjusted rate ratio of 1.48 (95% CI 1.45–1.51), as well as a higher overall risk of GDM, with an age adjusted rate ratio of 1.17 (95% CI 1.16–1.18) *p* < 0.01, compared to females living in the urban areas. There was an increasing trend over time.

The data from Database 1 regarding all cases of DM in pregnancy were divided into three age groups, each presenting certain peculiarities. The first category comprises adolescents and very young adults. This category is at high risk from a social point of view with regard to the education of the mother and the infant, as well as from a medical point of view with regard to adherence to treatment and recommendations. Although there are not many reported cases, it is important to bear in mind that this is a vulnerable group requiring special attention from social services as well as the medical system. Specific medical recommendations and follow-ups are necessary to ensure the best possible pregnancy outcome and postnatal care.

The second group, aged between 20 and 39 years, represents the majority of cases; the females from this group are of childbearing age. The last group, aged 40–49 years, is another high-risk category in which GDM is prevalent and where there are frequent pregnancy complications, including spontaneous abortions, GDM, gestational hypertension, preeclampsia and so on. This group also has a higher risk of adverse pregnancy outcomes, including premature birth, neonatal intensive care unit admissions and lower Apgar scores [34]. This group also has a higher risk of presenting comorbidities such as being overweight and obese, and it is the group in which artificial reproductive techniques are most commonly used. This group is also at higher risk of postpartum depression and requires a multidisciplinary approach to treatment and postpartum care [35]. Patients in the 10–14 age group in the two high-risk categories who received insufficient prenatal care, as well as socially vulnerable patients, although few were reported in this eleven-year period, still exist in real-life practice, indicating and signalling the fact that there are adolescents who could benefit from improved prenatal care. The highest rate of pregnant patients who received insufficient prenatal care was recorded in the 20–39 years age group (prevalence ranging from 0‰ to 0.50‰), followed by the 15–19 years age group. The numbers in these categories reached a peak of prevalence in 2017, followed by a 50% decrease in the numbers in 2024. Also, these age subgroups have reached the majority in the socially vulnerable group, leading to unsuitable prenatal care.

Regarding the urban–rural distribution of these high-risk pregnancies, the pregnant patients in the rural areas have a higher rate of insufficient prenatal care that ranges from 81.81% in 2014 to a slight decrease to 74.19% in 2024. The rates of socially vulnerable pregnancies also have a predominant rural distribution: 75% in 2014, an inverted ratio in favour of the urban area in 2016 and 2017, an equalized ratio in 2019 between rural and urban areas, and a similar distribution in 2024. By analyzing the particularities of these patients, pregnant patients who received insufficient prenatal care and socially vulnerable patients, the most vulnerable categories are adolescents and young adults, respectively, living in rural areas. We can conclude that there are measures that need to be taken in order to improve the care of defenceless females: improving the healthcare (preconceptionally, prenatally and also in the postpartum period), enhancing the medical knowledge, especially of the high-risk population, revising health policies and involving more and more social services. In addition, there is a critical need to emphasize the implications of underreporting, such as masking the true burden of DM in pregnancy, weakening health surveillance and policy planning, and altering preventive measures. Moreover, underreporting jeopardizes public safety, patients’ outcomes, resource planning, legal compliance and professional integrity.

### 4.1. Preventive Strategies

Following these observations, we would like to point out several aspects that require improvement. Firstly, primary care should include T2DM and GDM prevention and preconceptional care for pregestational DM via general practitioners and obstetricians, aligning with the ADA recommendations.

### 4.2. Preconceptional Care and DM in Pregnancy Complications Prevention

Preconceptional counselling should begin at puberty and continue for all people of childbearing age and DM, alongside with effective contraception and family planning focusing on educating the people about the importance of a proper glycemic control, reaching to an ideal a HbA1c value of <6.5%, in order to prevent the major DM complications in pregnancy, such as macrosomia, congenital anomalies, preterm birth or preeclampsia [35]. Patients with a history of GDM should be screened preconceptionally for DM and should receive preconception care in order to diagnose and treat hyperglycemia, therefore reducing the risk of congenital malformations [35]. The attention should focus on physical activity, nutrition, DM self-care education, as well as screening for DM comorbidities and complications, such as diabetic retinopathy or nephropathy. Patients with pregestational diabetes should be informed regarding the risk of developing and/or progression of diabetic retinopathy and should undergo dilated eye examinations ideally before pregnancy, every trimester and 1 year postpartum [36].

### 4.3. Lifestyle Optimization

If diagnosed, the first step in managing GDM is represented by medical nutrition therapy. To prevent GDM complications, adequate glycemic control is desired. Prenatal care should include healthy lifestyle counselling; more specifically, it should include information about an adequate diet that promotes healthy fetal development and optimal glycemic control, and it should advocate for physical activity, that is, at least 150 min of aerobic activity of moderate intensity weekly [37]. Concerning nutrition, the recommendation is not to exclude carbohydrates. The proposed diet should include at least 175 g of carbohydrates, followed by at least 71 g of protein and 28 g of fibre per day [38]. Restricting carbohydrate intake is often accompanied by increased fat intake, which aggravates maternal insulin resistance, amplifies lipolysis, increases free fatty acid levels [39], and is responsible for fetal overgrowth [40].

### 4.4. DM in Pregnancy Monitoring and Treatment

Monitoring pregnancies with DM should focus on a multidisciplinary approach, respectively, an active collaboration between obstetricians, diabetologists and general practitioners. Obstetrical monitoring consists of fetal monitoring, including fetal growth curve, the presence or absence of polyhydramnios and fetal wellbeing, as well as maternal wellbeing. It also involves identifying possible maternal complications such as gestational hypertension [41]. Glycemic control can be achieved using the classical method, respectively, self-monitoring blood glucose (SMBG), which involves measuring fasting blood glucose concentrations daily, as well as before each meal and one or two hours after eating [41]. Alternatively, continuous glucose monitoring systems (CGMS) can be used [42]. The latter have already proven their efficacy in monitoring T1DM during pregnancy in the CONCEPTT trial [43]. Unfortunately, CGMS are not used on a large scale for pregnancies with T2DM or GDM, despite their potential to obtain a more detailed picture of the glycemic variability. It should be noted that CGMS offer information on nocturnal glycemic values and could facilitate the timely initiation of insulin therapy for GDM, thereby avoiding pregnancy complications caused by exposure to hyperglycemia in utero [44,45,46]. A study by Alfadhli et al. [47] found that pregnancies with GDM monitored by CGMS required smaller insulin doses than those monitored by SMBG.

In addition to medical nutrition therapy, insulin therapy is the second-line treatment for GDM and the first-line treatment for pregestational DM [48,49]. Optimal insulin therapy could prevent hyperglycemia-related complications from the first trimester, when hyperglycemia can negatively affect the normal development of the embryo, resulting in congenital malformations, to the last trimester of pregnancy, when it can prevent macrosomia, neonatal complications and even stillbirth. In this regard, hybrid closed-loop insulin delivery systems, the newest technology, could improve pregnancy outcomes in women with T1DM. This system, consisting of CGMS and insulin pumps, can be started preconceptionally to improve glycemic values and avoid changing therapy during the first trimester [50].

### 4.5. Improving the Present Medical Knowledge

This should be achieved by introducing health education in schools and running nutrition awareness campaigns. National health policies should also be revised. Patients at risk of developing T2DM should be systematically screened according to the guidelines, using fasting blood glucose levels and HbA1c. This category includes overweight or obese adults with at least one of the following risk factors: a history of CV disease; belonging to a high-risk race, ancestry or ethnicity; having a first-degree relative diagnosed with DM; hypertension; physical inactivity; polycystic ovary syndrome; or other pathology associated with insulin resistance, such as metabolic dysfunction or acanthosis nigricans. It also includes patients with high-density lipoprotein (HDL) cholesterol levels of less than 35 mg/dL, patients with a history of GDM, or who are at least 35 years old. Patients in high-risk groups, such as those with human immunodeficiency virus infection or a history of pancreatitis, are also included [2]. Screening should also not exclude children or adolescents at risk of developing prediabetes or T2DM, beginning with the onset of puberty or at the age of 10. This category includes overweight or obese children and adolescents whose mothers presented GDM or pregestational DM, or who have a first- or second-degree family member with T2DM, or who belong to a high-risk ethnic group, or who present signs of insulin resistance or insulin resistance-associated pathologies (e.g., polycystic ovary syndrome, acanthosis nigricans, dyslipidemia, hypertension), or who had a birth weight that was large or small for gestational age [2]. Adults and children/adolescents at high-risk of developing T2DM could certainly benefit from prediabetes and DM screening and timely intervention involving lifestyle adjustments, nutritional education and the initiation of physical activity.

### 4.6. Postpartum Care

In our study, the majority of DM cases in pregnancy were represented by GDM. Therefore, postpartum care should include lifelong care for these patients, as well as screening every 1–3 years for the early detection of prediabetes and T2DM. This screening should be carried out at 4–12 weeks with a 75 g oral glucose tolerance test. Patients with a history of GDM who are overweight or obese should be counselled and educated to embrace a healthy lifestyle and lose weight. If prediabetes is diagnosed in these patients, thorough lifestyle interventions should be implemented and metformin administered, if necessary, to prevent the development of DM [37].

### 4.7. Improving the Social Services’ Involvement

Social services should monitor high-risk pregnancies involving women at risk due to social issues or inequalities, and those who could receive inadequate prenatal care. This would ensure that these women have access to medical care and adhere to recommendations and treatment. The social services department should be involved in caring for vulnerable women and should be an institution that is easy to approach. It should offer an empathetic and safe environment for women at risk in order to achieve the best possible outcomes for pregnancies and for the future development of their children.

**Figure 4:**
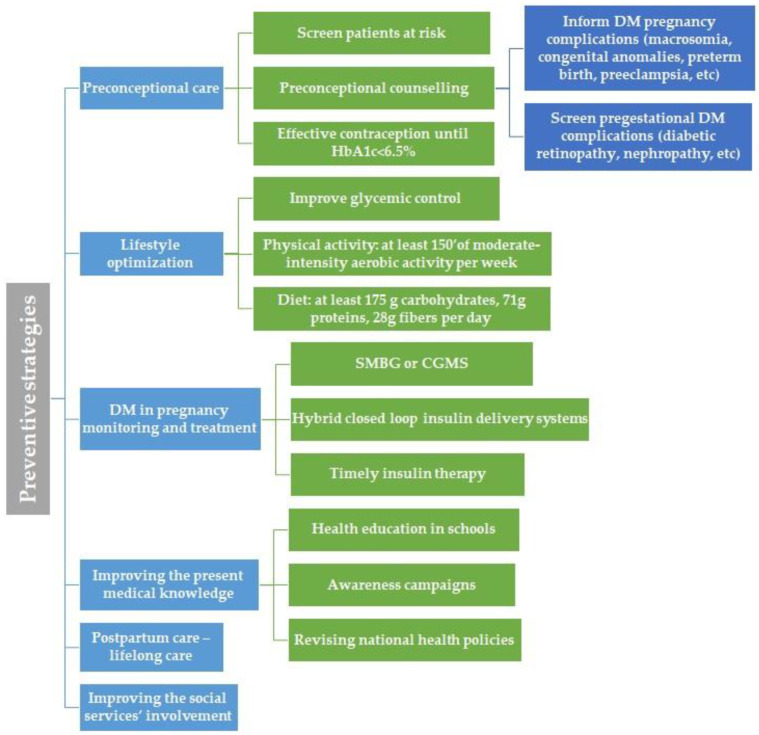
Main preventive strategies proposal for pregnant women with DM in Romania.

Finally, we created an illustration that summarizes the main proposed preventive strategies for improving care of pregnant women with DM in Romania (Figure 4).

### 4.8. Future Directions

Firstly, clear and homogeneous criteria for GDM diagnosis should be established worldwide, along with universally accepted cut-offs for exact differential diagnosis between pregestational DM and GDM. Secondly, to gather all the data on DM in pregnancy in Romania and to gain a more complete picture, the reporting system requires substantial and continuous improvement. There is a gap in the reporting system from the medical units associated with the National Insurance House that needs to be addressed. The National Public Health Department would also benefit from data reported from the private medical sector. Furthermore, future efforts should aim to harmonize data collection between the two national databases to avoid duplication and underestimation. Improved digitalization and standardized ICD coding across all reporting levels could significantly increase accuracy and comparability with international statistics. Collaboration between general practitioners and public and private medical units must also be strengthened to ensure accurate reporting of cases. Another area for future research could be the study of different social, economic or demographic factors that could influence the occurrence of DM in pregnancy among Romanian patients.

Another important area for development is the integration of socio-economic and environmental factors into surveillance systems. Current findings show that adolescents, women of advanced maternal age, and those from rural areas or with insufficient prenatal care remain highly vulnerable. Personalized interventions for these groups, supported by both healthcare and social services, should be prioritized.

Moreover, prospective cohort studies and multicenter collaborations at national and regional levels could provide deeper insights into the long-term maternal and offspring outcomes of DM during pregnancy. This would enable more effective prevention strategies, timely interventions and evidence-based health policies that address both medical and social inequities.

Finally, improving the healthcare system involves the need for changing the law; healthcare providers and legislators should be focusing on:

- Solving the underreporting issue by establishing a functional system or registry for reporting the DM in pregnancy cases;
- Achieving DM subtype diagnosis, monitoring, treatment, peripartum and postpartum care standardization by integrating the existing international guidelines into the local and, respectively, national context;
- Improving the medical knowledge of the Romanian population by introducing age-adapted healthcare classes from primary school to high school, running awareness campaigns, nutritional workshops, organizing or supporting local or regional physical activity events;
- Ensuring a better availability of medical and social support for vulnerable categories (adolescents, young adults, patients from rural areas, socio-economically unstable families) in order to increase the addressability of these categories, regulating a multidisciplinary approach of such cases: general practitioner, diabetologist, obstetrician, psychologist and social services worker.

### 4.9. Strengths and Limitations

The main strength of the present study lies in the data regarding DM in pregnancy, as well as in pregnant females who received insufficient prenatal care or who presented social issues in Romania in the last decade. The central limitation is the national reporting system, which the data highlights as having significant gaps. This system likely underestimates the true burden of DM in pregnancy compared to international numbers. Some public institutions might have failed to classify the disease according to the ICD system, and some patients were monitored and gave birth exclusively in private medical units that do not report to the National Public Health Department. Another limitation of the present study is represented by the lack of data on socio-economic and demographic risk factors that could characterize Romanian patients.

## 5. Conclusions

Despite a general downward trend in the total number of pregnancies, the prevalence of DM, particularly GDM, has steadily increased, mirroring global trends and emphasizing the growing impact of metabolic disorders on maternal and fetal health. Vulnerable populations, including adolescents, females of advanced maternal age, and those with inadequate prenatal care or social risk factors, remain at high risk for adverse outcomes and require targeted interventions. An essential approach is a multifaceted, collaborative one, which includes enhanced screening protocols, comprehensive preconceptional and prenatal counselling, the integration of new technologies such as CGMS or hybrid closed-loop insulin delivery systems, and widespread education on nutrition and physical activity. Social services should play an active role in supporting high-risk women to ensure equitable access to healthcare. Furthermore, we must emphasize the need for policy reform, community-based prevention strategies, and early and proactive intervention. Only through sustained and coordinated efforts can we reduce the burden of DM in pregnancy and secure better health for mothers and future generations in Romania.

## Figures and Tables

**Table 1:** Total number and DM type distribution of DM complicated pregnancies from Romania between 2014 and 2024– as reported by Database 1.

Year	Reported Pregnancies (*n*)	Pregnancies Complicated by DM (*n, Prevalence ‰*)	DM Type (*n*)		
			Type 1	Type 2	GDM
2014	129,723	132, 1.02	27	18	87
2015	129,729	137, 1.06	23	20	94
2016	128,932	181, 1.40	31	16	134
2017	120,851	322, 2.66	39	17	266
2018	114,554	353, 3.08	44	26	283
2019	109,210	347, 3.18	43	24	280
2020	102,063	194, 1.90	17	8	169
2021	97,964	244, 2.49	35	10	199
2022	91,006	276, 3.03	23	13	240
2023	85,932	234, 2.72	37	10	187
2024	83,480	251, 3.01	34	17	200

DM—Diabetes mellitus; GDM—Gestational diabetes mellitus.

**Table 2:** Age group distribution of DM in pregnancy cases from Romania between 2014 and 2024, as reported by Database 2.

Age Group (*Years*)	2014 *(%)*	2015 (*%*)	2016 (*%*)	2017 (*%*)	2018 (*%*)	2019 (*%*)	2020 (*%*)	2021 (*%*)	2022 (*%*)	2023 (*%*)	2024 (*%*)
10–14	0	0.79	0	0	0	0.43	1.07	1.01	0.28	0.24	0
15–19	0	1.57	1.12	0.53	1.51	0	2.14	0	1.69	0.95	1.62
20–39	88.07	92.91	93.85	95.26	92.46	95.32	89.64	91.95	90.99	91.45	88.46
40–54	11.93	4.72	5.03	4.21	6.03	4.26	7.14	7.05	7.04	7.36	9.92
Total cases	109	127	179	190	199	235	280	298	355	421	494

**Table 3:** DM cases distribution in pregnancies from Romania between 2014 and 2024 by environmental settlement as reported by Database 1.

Year	DM in Pregnancy Cases (*n*)	Urban Distribution of Patients (*%*)	Rural Distribution of Patients (*%*)
2014	132	64.40	35.60
2015	137	60.58	39.42
2016	181	66.85	33.15
2017	322	68.94	31.06
2018	353	70.25	29.75
2019	347	63.69	36.31
2020	194	58.76	41.24
2021	244	59.84	40.16
2022	276	57.25	42.75
2023	234	49.15	50.85
2024	251	56.18	43.82

DM—diabetes mellitus.

**Table 4:** Distribution of cases of DM in pregnancy from Romania between 2014 and 2024 after the environmental settlement, as reported by Database 2.

Year	DM in Pregnancy Cases (*n*)	Urban Number of Patients (*%*)	Rural Number of Patients (*%*)
2014	109	52.29	47.71
2015	127	64.57	35.43
2016	179	63.69	36.31
2017	190	56.84	43.16
2018	199	62.81	37.19
2019	235	66.38	33.62
2020	280	66.43	33.57
2021	298	64.43	35.57
2022	355	60.85	39.15
2023	421	60.81	39.19
2024	494	58.10	41.90

DM—diabetes mellitus.

**Table 5:** DM type distribution of cases of DM in pregnancies from Romania between 2014 and 2024 by age groups (10–19, 20–39 and 40–49 years), as reported by Database 1.

DM Type	Age Group	2014 (%)	2015 (%)	2016 (%)	2017 (%)	2018 (%)	2019 (%)	2020 (%)	2021 (%)	2022 (%)	2023 (%)	2024 (%)
T1DM	10–19	1.52	0.73	1.10	0.62	0.28	2.31 *	1.55	0.41	0	0.43	0.80
	20–39	18.18	15.33	14.92	10.25	11.05	9.22	7.22	12.70	6.88	15.38	10.36
	40–49	0.76	0.73	1.10	1.24	1.13	0.86	0	1.23	1.09	0	2.79
T2DM	10–19	0	0	0	0.31	0.85	0	0	0	0.36	0.43	0
	20–39	12.12	20.44	8.84	4.35	5.67	6.05	4.12	3.28	4.71	3.42	5.58
	40–49	1.52	0.73	0	0.62	0.85	0.86	0	0.82	0	0.43	0.80
GDM	10–19	3.79	0.73	1.66	1.24	2.55	0.58	0.52	2.46	1.45	2.14 *	1.59
	20–39	59.09	59.12	66.85	74.53	73.37	74.35	78.87	71.31	80.43	71.79	70.92
	40–49	3.03	8.76	5.52	6.83	4.25	5.76	7.73	7.79	5.07	5.98	7.17

DM—diabetes mellitus; * There were two cases of DM in the age group 10–14 years: one T1DM case in 2019 and one GDM case in 2023.

**Table 6:** Distribution of cases of pregnancies from Romania between 2014 and 2024 that received insufficient prenatal care and socially vulnerable patients, as reported in Database 1.

Risk Group	Age Group (*Years*)	2014 (*n*)	2015 (*n*)	2016 (*n*)	2017 (*n*)	2018 (*n*)	2019 (*n*)	2020 (*n*)	2021 (*n*)	2022 (*n*)	2023 (*n*)	2024 (*n*)
Insufficient prenatal care	10–14	0	0	0	0	1	0	1	0	0	0	0
	15–19	3	9	11	24	18	10	24	19	8	11	6
	20–39	8	21	38	55	41	34	0	26	11	26	23
	40–49	0	2	3	2	3	2	32	2	1	1	2
Socially vulnerable patients	10–14	0	0	0	0	0	0	0	0	0	1	0
	15–19	1	3	5	1	4	0	1	0	1	1	1
	20–39	3	0	9	7	3	2	5	1	3	1	6
	40–49	0	0	2	0	0	0	0	1	0	0	1

**Table 7:** Distribution of cases of pregnancies from Romania between 2014 and 2024 that received insufficient prenatal care and socially vulnerable patients by environmental settlement, as reported in Database 1.

Risk Group	Area	2014 (*n*)	2015 (*n*)	2016 (*n*)	2017 (*n*)	2018 (*n*)	2019 (*n*)	2020 (*n*)	2021 (*n*)	2022 (*n*)	2023 (*n*)	2024 (*n*)
Insufficient prenatal care	Urban	2	8	12	19	13	12	8	14	7	9	8
	Rural	9	24	40	62	50	34	49	33	13	2	23
Socially vulnerable patients	Urban	1	1	11	5	3	1	2	2	0	1	4
	Rural	3	2	5	3	4	1	5	0	4	2	4

**Table 8:** Correlations between risk factors and total number of pregnancies, total number of DM cases in pregnancy, the number of cases of T1DM in pregnancy, T2DM in pregnancy and GDM.

Group	Urban Area	Rural Area	Socially Vulnerable Patients	Patients Who Received Insufficient Prenatal Care
Total number of pregnancies	*p* = 0.450	*p* = 0.013	*p* = 0.301	*p* = 0.432
DM in pregnancy cases	*p* = 0.00	*p* = 0.007	*p* = 0.989	*p* = 0.199
T1DM in pregnancy cases	*p* = 0.010	*p* = 0.091	*p* = 0.703	*p* = 0.368
T2DM in pregnancy cases	*p* = 0.391	*p* = 0.989	*p* = 0.935	*p* = 0.773
GDM cases	*p* = 0.022	*p* = 0.172	*p* = 0.817	*p* = 0.337

DM—Diabetes mellitus, T1DM—Type 1 diabetes mellitus, T2DM—Type 2 diabetes mellitus, GDM—Gestational diabetes mellitus.

## Data Availability

Data available on request from the authors and from the Romanian National Public Health Institute, due to the fact that this is the institution’s property.

## Abbreviations

The following abbreviations are used in this manuscript:

ADA	American Diabetes Association
CGMS	Continuous glucose monitoring system
CI	Confidence interval
CV	Cardiovascular
DM	Diabetes mellitus
GDM	Gestational diabetes mellitus
HbA1c	Glycated hemoglobin
HDL	High-density lipoprotein
ICD	International Classification of Diseases
SMBG	Self-monitoring blood glucose
T1DM	Type 1 diabetes mellitus
T2DM	Type 2 diabetes mellitus
